# Comparison of in vitro antileukemic activity of obatoclax and ABT-737

**DOI:** 10.1007/s13277-016-4943-z

**Published:** 2016-02-15

**Authors:** Małgorzata Opydo-Chanek, Lidia Mazur

**Affiliations:** Department of Experimental Hematology, Jagiellonian University, Gronostajowa 9, 30-387 Krakow, Poland

**Keywords:** BH3 mimetics, Obatoclax, ABT-737, Anticancer agents, Antileukemic activity

## Abstract

Obatoclax and ABT-737 belong to a new class of anticancer agents known as BH3-mimetics. These agents antagonize the anti-apoptotic members of Bcl-2 family. The Bcl-2 proteins modulate sensitivity of many types of cancer cells to chemotherapy. Therefore, the objective of the present study was to examine and compare the antileukemic activity of obatoclax and ABT-737 applied alone, and in combination with anticancer agent, mafosfamide and daunorubicin. The in vitro cytotoxic effects of the tested agents on human leukemia cells were determined using the spectrophotometric MTT test, Coulter electrical impedance method, flow cytometry annexin V–fluorescein/propidium iodide assay, and light microscopy technique. The combination index analysis was used to quantify the extent of agent interactions. BH3 mimetics significantly decreased the leukemia cell viability and synergistically enhanced the cytotoxic effects induced by mafosfamide and daunorubicin. Obatoclax affected the cell viability to a greater degree than did ABT-737. In addition, various patterns of temporary changes in the cell volume and count, and in the frequency of leukemia cells undergoing apoptosis, were found 24 and 48 h after the tested agent application. ABT-737 combined with anticancer agents induced apoptosis more effectively than obatoclax when given in the same combination regimen. The results of the present study point to the different antileukemic activities of obatoclax and ABT-737, when applied alone, and in combination with anticancer agents. A better understanding of the exact mechanisms of BH3 mimetic action is of key importance for their optional use in cancer therapy.

## Introduction

Leukemia is a hematological malignancy that is characterized by the uncontrolled accumulation of pathological white blood cells. Currently, chemotherapy is a major pharmacological approach in the treatment of leukemia [1]. To improve the efficacy of anticancer agents and increase the therapeutic index, many strategies have been designed. One of the approaches for leukemia treatment is the development of BH3 mimetic compounds [2, 3].

The BH3 mimetics are small molecule antagonists of the anti-apoptotic members of Bcl-2 protein family. These agents mimic the structure and function of BH3-only proteins and interact with anti-apoptotic Bcl-2 proteins at their BH3-binding hydrophobic groove [4]. Members of the Bcl-2 family are thought to be one of the crucial mediators involved in mitochondrial cell death pathway [5]. Anti-apoptotic members of Bcl-2 family are frequently overexpressed in almost all types and subtypes of leukemia, indicating the importance of these molecules in disease pathogenesis and treatment failure [6]. The inhibition of anti-apoptotic proteins of Bcl-2 family by BH3 mimetics is an attractive strategy for restoring apoptotic process in leukemia cells or making these cells more susceptible to the action of anticancer agents.

Among the BH3 mimetics discovered to date, obatoclax (Obat) and ABT-737 are promising anticancer agents [2, 5, 7, 8]. Obatoclax (2-[5(3,5-dimethyl-1H-pyrrol-2-ylmethylene)-4-methoxy-5H-pyrrol-2-yl]-1H-indole) is a synthetic indol bipyrrol derivative of bacterial prodiginines, developed by Gemin X Biotechnologies. ABT-737 was developed by Abbott Laboratories using a combination of approaches, including NMR-based screening, parallel synthesis and structure-based design [9]. Currently, obatoclax, and ABT-263, an orally available analog of ABT-737, are under investigation in clinical trials as a novel anticancer agent for hematologic and lymphoid malignancies [7, 10–12]. Nevertheless available information on anticancer potential of Obat and ABT-737 is still scarce. Thus, it is of great interests to precisely defined antileukemic efficacy of obatoclax and ABT-737, when applied alone and also in combination with other anticancer agents, e.g. mafosfamide and daunorubicin. Mafosfamide (MAF), a novel cyclophosphamide analog, belongs to oxazaphosphorines, a group of alkylating agents [13]. Daunorubicin (DAU), an anthracycline antibiotic, is widely used in anticancer therapy [14]. The purpose of the present in vitro studies was to determine and compare the antileukemic activity of obatoclax and ABT-737, given alone and in combination with mafosfamide or daunorubcin. The influence of the tested agents on the cell viability, volume and count as well as apoptosis-induction was determined.

## Materials and Methods

### Chemicals

Obatoclax mesylate and ABT-737 were purchased from Selleck Chemicals (Munich, Germany), dissolved in DMSO and stored as 5 mM stock solutions at −20 °C. Mafosfamide cyclohexylamine salt was kindly provided by Dr Ulf Niemeyer (NIOMECH, Bielefeld, Germany) and dissolved in aqua pro injectione (Polpharma, Poland) directly before treatment of cells. Daunorubicin was purchased from Sigma Aldrich (St. Louis, MO, USA) and stored as 0.5 mM stock solutions in aqua pro injectione at −20 °C. RPMI 1640 medium and fetal calf serum were from GIBCO BRL Life Technologies (Gaithersburg, MD, USA). L-glutamine, antibiotic antimycotic solution (AAS), dimethyl sulfoxide (DMSO), (4,5-dimethylthiazol-2-yl)-2,5-diphenyl-tetrazolium bromide (MTT) and acridine orange were purchased from Sigma Aldrich. FITC Annexin V Apoptosis Detection Kit I was obtained from BD Pharmingen (BD Biosciences, San Diego, CA).

### Cell lines and culture

Human promyelocytic leukemia HL-60 cells and human promonocytic leukemia U-937 cells were purchased from American Type Culture Collection (Rackville, USA). Human acute myeloblastic leukemia ML-1 cells and human acute lymphoblastic leukemia MOLT-4 cells were obtained from European Collection of Cell Cultures (Salisbury, UK). HL-60, U-937, ML-1, and MOLT-4 cells were maintained in RPMI 1640 medium supplemented with 10 % fetal calf serum, 2 mM L-glutamine and AAS containing 20 U of penicillin, 20 mg streptomycin and 0.05 mg amphotericin B. Cells were passaged every third day. The cells grew exponentially at 37 °C in an atmosphere of 5 % CO_2_ in air (HERAcell incubator, KendroLab). Leukemia cells were seeded in 24-well plates, at a density of 15 × 10^4^ cells/mL, prior to performing each experiment.

### Cell viability assay

The influence of Obat and ABT-737 given alone or in combination with MAF or DAU on the leukemia cell viability was analyzed according to the procedure of the spectrophotometric MTT assay, previously described by Mazur et al. [15]. The extent of MTT conversion to formazan in the cells reflects their viability. The values of absorbance of the obtained formazan solutions were measured at a wavelength of 570 nm using a Pharmacia Ultrospec III spectrophotometer (Pharmacia), and were expressed as percentage values of the control. HL-60, U-937, ML-1, and MOLT-4 leukemia cells were treated with Obat or ABT-737, at the concentration range from 0.1 to 10 μM. The leukemia cells were exposed to MAF at the concentration range from 1 to 100 μM , and DAU from 0.01 to 1 μM. After a 48-h exposure of the human leukemia cells to the tested agents, the IC_25_ and IC_50_ values were calculated from the dose–response curves as the agent concentrations necessary for 25 and 50 % inhibition of the cell viability**,** respectively. The IC_25_ or IC_50_ value for two BH3 mimetics and IC_25_ value for both mafosfamide and daunorubicin, was chosen to investigate effects of the combined action of these agents on leukemia cells. The controls consisted of untreated and DMSO-treated leukemia cells.

### Determination of cell volume, count, and death

Temporary changes occurring in the volume and count of HL-60 leukemia cells, and also the frequency of cells undergoing apoptosis, were determined 24 and 48 h after the application of Obat and ABT-737 alone, and in combination with MAF or DAU. HL-60 leukemia cells were treated with 0.5 μM of Obat, 2.5 μM of ABT-737, 20 μM of MAF, and 0.15 μM of DAU. The used concentrations correspond to the IC_25_ values of each tested agent.

#### Coulter counter measurements

The HL-60 leukemia cell volume and count were analyzed using a Z2 Coulter counter (Beckman Coulter, USA) as previously described in detail by Mazur et al. [15]. The mean cell volume and also the cell count were determined at a range 832–7346 fL, using Z2 AccuComp software (Beckman Coulter, USA).

#### Annexin V-FITC/PI assay

Dual staining of HL-60 leukemia cells with fluoresceinated annexin V (annexin V-FITC) and propidium iodide (PI) was performed according to the manufacturer’s instruction. Briefly, the cells were washed twice with cold PBS and resuspended in 100 μL of binding buffer. Then 5 μL of annexin V-FITC and 5 μL of PI staining solution was added to the cell suspension, containing approximately 1.5 × 10^5^ cells, and the cells were incubated in the dark for 15 min, at room temperature. Following the incubation, 400 μL of binding buffer was added to each tube. Cell samples were placed on ice, away from light, and FITC and PI fluorescence was immediately measured using FACSCalibur flow cytometer (Becton Dickinson). The frequency of early apoptotic cells, and also the frequency of late apoptotic and necrotic cells, were determined using WinMDI 2.8 software.

#### Microscopy analysis of leukemia cell morphology

HL-60 cell suspension, eventually diluted in PBS, containing approximately 1 × 10^5^ cells, was added into a cytospin chamber and centrifuged at 1000 rpm (MPW-350R centrifuge, Med. Instruments) for 6 min, at 4 °C. After air drying, the prepared cytospins were fixed in methanol for 15 min, at room temperature. The cytospins were stained with an acridine orange solution (Sigma Aldrich) for 3 min. A working solution of acridine orange was prepared from 0.1 % aqueous stock solution (2 parts of the stock solution and 30 parts of PBS, pH = 6.8). After staining, the cytospins were rinsed in PBS and mounted under a coverslip in a drop of PBS. The prepared cytospins were examined under 400× magnification, using a Jenaval epifluorescent microscope (Carl Zeiss, Germany). Based on the morphology of HL-60 cells, the apoptotic index was calculated as the percentage of apoptotic cells identified among 9000 leukemia cells (3000 cells per slide).

### Calculation of synergy and statistical evaluation

The obtained results were confirmed by three independent experiments carried out in duplicate or triplicate. All the data are presented as the mean value ± standard deviation (SD). The agent interactions were analyzed using CompuSyn software (ComboSyn Inc., USA). Combination index (CI) values were calculated according to the Chou and Talalay mathematical model for drug interactions [16]. CI < 1.0 represents synergy, CI = 1.0 indicates an additive effect whereas CI > 1.0 implies antagonism. The statistical significance for the data was evaluated by an analysis of variance and Duncan’s new multiple range test. *P* values *<* 0.05 were considered statistically significant.

## Results

### Influence of BH3 mimetics on leukemia cell viability

**Fig. 1:**
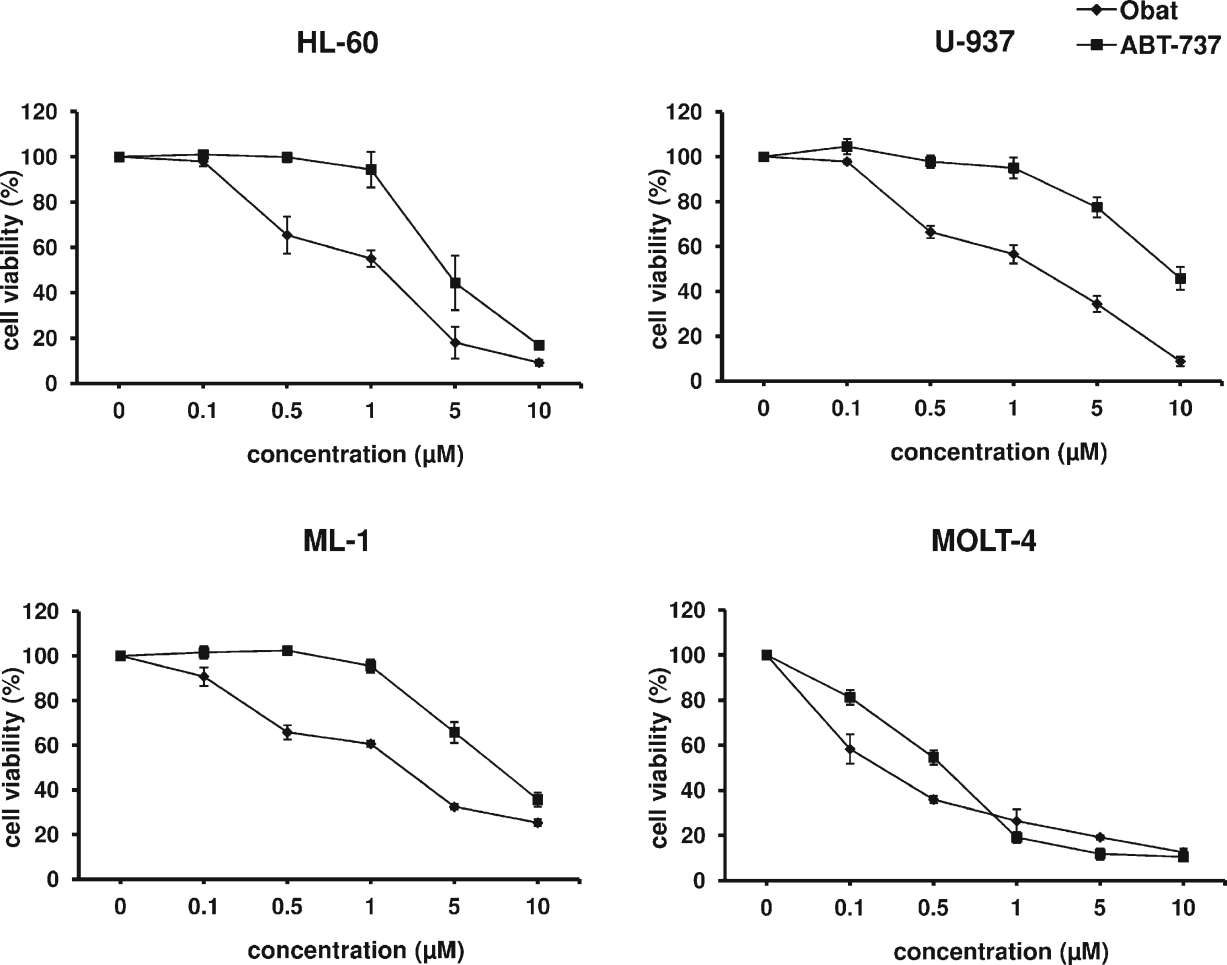
The leukemia cell viability determined at 48 h after obatoclax and ABT-737 application, using MTT assay. The data are presented as the mean ± SD from three independent experiments carried out in triplicate

The cell viability is a critical factor for evaluation of cell response to the action of cytotoxic agents. As shown in Figure 1, the application of obatoclax and ABT-737 resulted in a dose-dependent decrease of the leukemia cell viability. Obat affected the cell viability to a greater degree than did ABT-737. Among the human leukemia cell lines, the lowest IC_50_ values for both BH3 mimetics were found for MOLT-4 cells. IC_50_ values for Obat and ABT-737 determined for HL-60 cells were approximately 10-fold higher compared to those calculated for MOLT-4 cells. BH3 mimetics appeared to be less active in U-937 and ML-1 cells than in HL-60 and MOLT-4 cells (Table 1).

**Table 1:** The IC_50_ values of obatoclax and ABT-737 calculated for the leukemia cell lines. Based on the obtained data using the in vitro MTT assay, the IC_50_ values were calculated from the dose–response curves. The data are presented as the mean ± SD

Cell line	HL-60	U-937	ML-1	MOLT-4
IC_50_ Obat (μM)	1.3 ± 0.15	1.7 ± 0.25	2.1 ± 0.15	0.13 ± 0.03
IC_50_ ABT-737 (μM)	4.35 ± 0.7	9.25 ± 0.4	8.3 ± 0.45	0.55 ± 0.02

### Combined effects of BH3 mimetic and MAF or DAU on leukemia cell viability

**Fig. 2:**
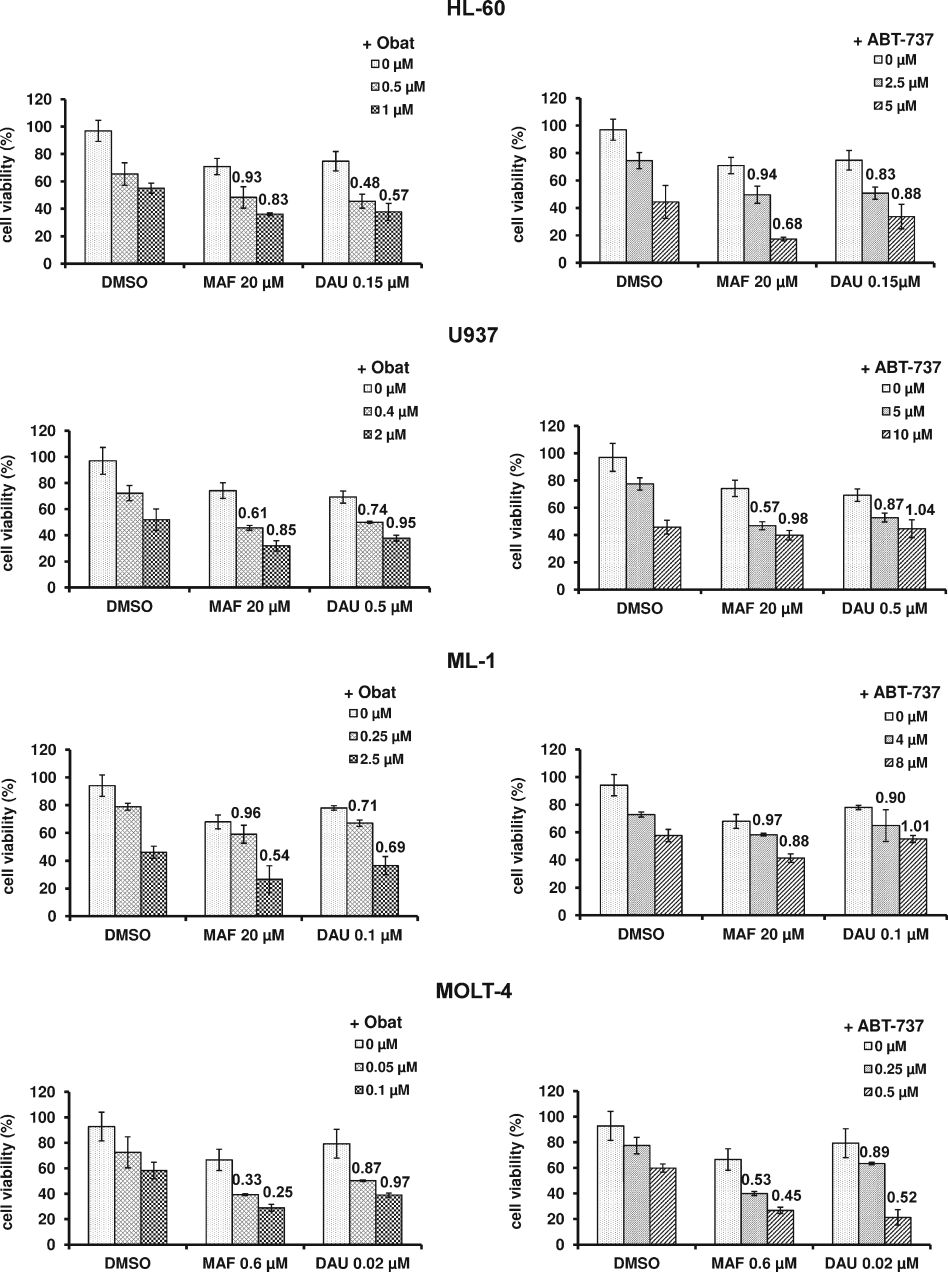
Effects of obatoclax and ABT-737 applied alone and in combination with mafosfamide or daunorubicin on the leukemia cell viability. HL-60, U-937, ML-1 and MOLT-4 cells were exposed to Obat or ABT-737, at the concentrations corresponding to the IC_25_ or IC_50_ value, in the absence or presence of MAF or DAU, given at a concentration corresponding to the IC_25_ value. The concentrations of the tested agents were calculated from the dose–response curves obtained for each leukemia cell line using MTT assay. The synergism between the tested agents was determined using the combination index analysis, at a non-constant ratio. The CI values, generated using the CompuSyn software according to the Chou–Talalay method, are plotted on the graph. The combination of BH3 mimetic with MAF or DAU is synergistic when CI < 1.0 and additive when CI = 1. The data are presented as the mean ± SD from three independent experiments carried out in triplicate

**Fig. 3:**
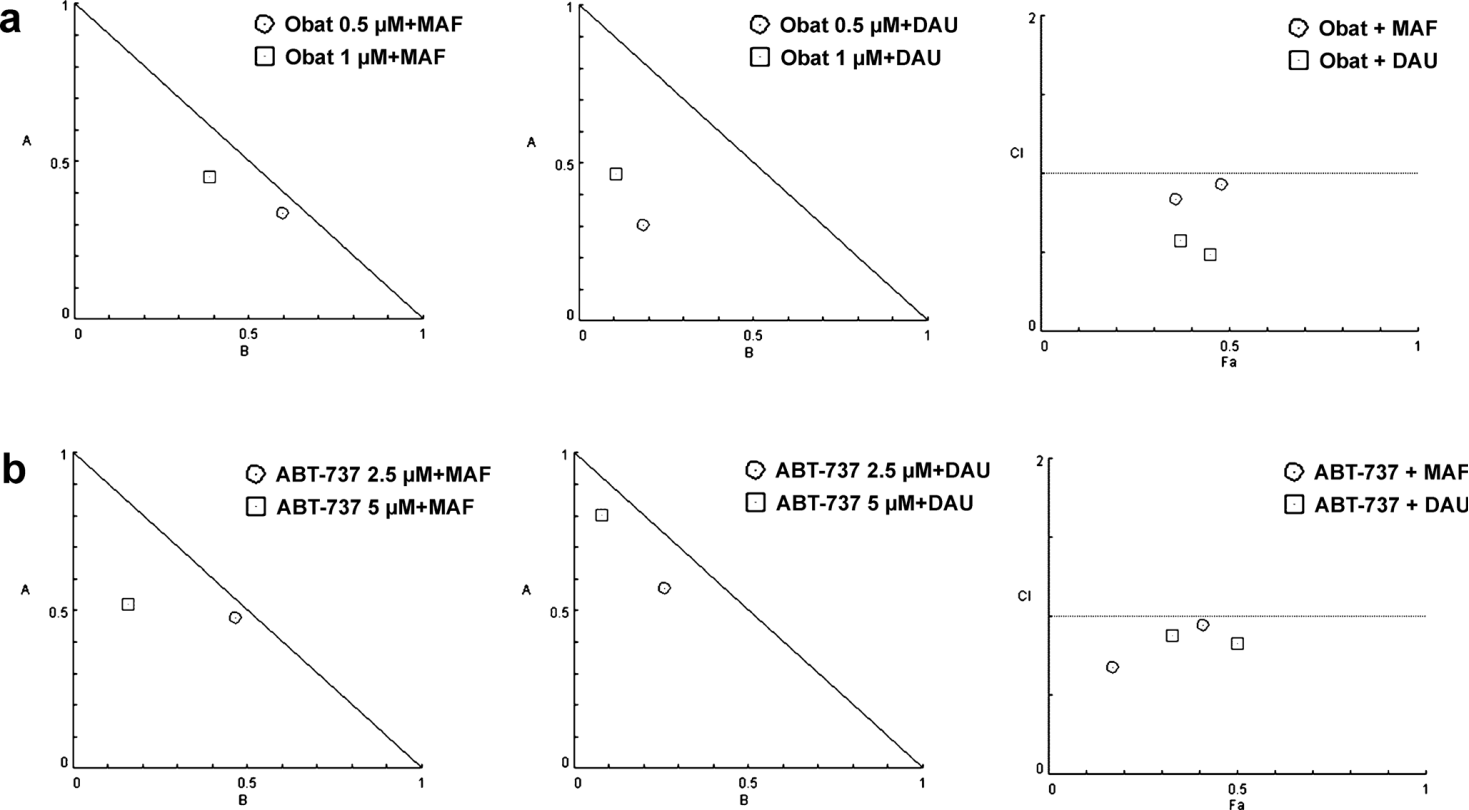
The CI values corresponding to the obatoclax or ABT-737 combinations with mafosfamide or daunorubicin were determined by isobologram analysis. A representative normalized isobolograms and fraction affected (Fa)-CI plots generated for HL-60 cells exposed to the action of Obat (**a**) and ABT-737 (**b**) in combination with MAF or DAU, graphically depict the interaction between the tested agents. *Points falling below the line* indicate synergism

To determine whether BH3 mimetics can enhance the cytotoxicity induced by mafosfamide or daunorubicin, the leukemia cells were exposed to the combined action of these agents. The cytotoxic effects of Obat, ABT-737, MAF, and DAU were measured using MTT assay and evaluated with CompuSyn software. The combined application of BH3 mimetic with MAF or DAU affected the leukemia cell viability to a higher degree than did each of the tested agents given alone (Fig. 2). Calculation of the CI values confirmed that the interactions between the tested agents were mostly synergistic in all leukemia cell lines (Figs. 2, 3). Additive effects were only observed in U-937 and ML-1 cells exposed to the combined action of ABT-737 and DAU, in the case when ABT-737 was given at the IC_50_ concentration.

### Changes in leukemia cell volume and count

**Fig. 4:**
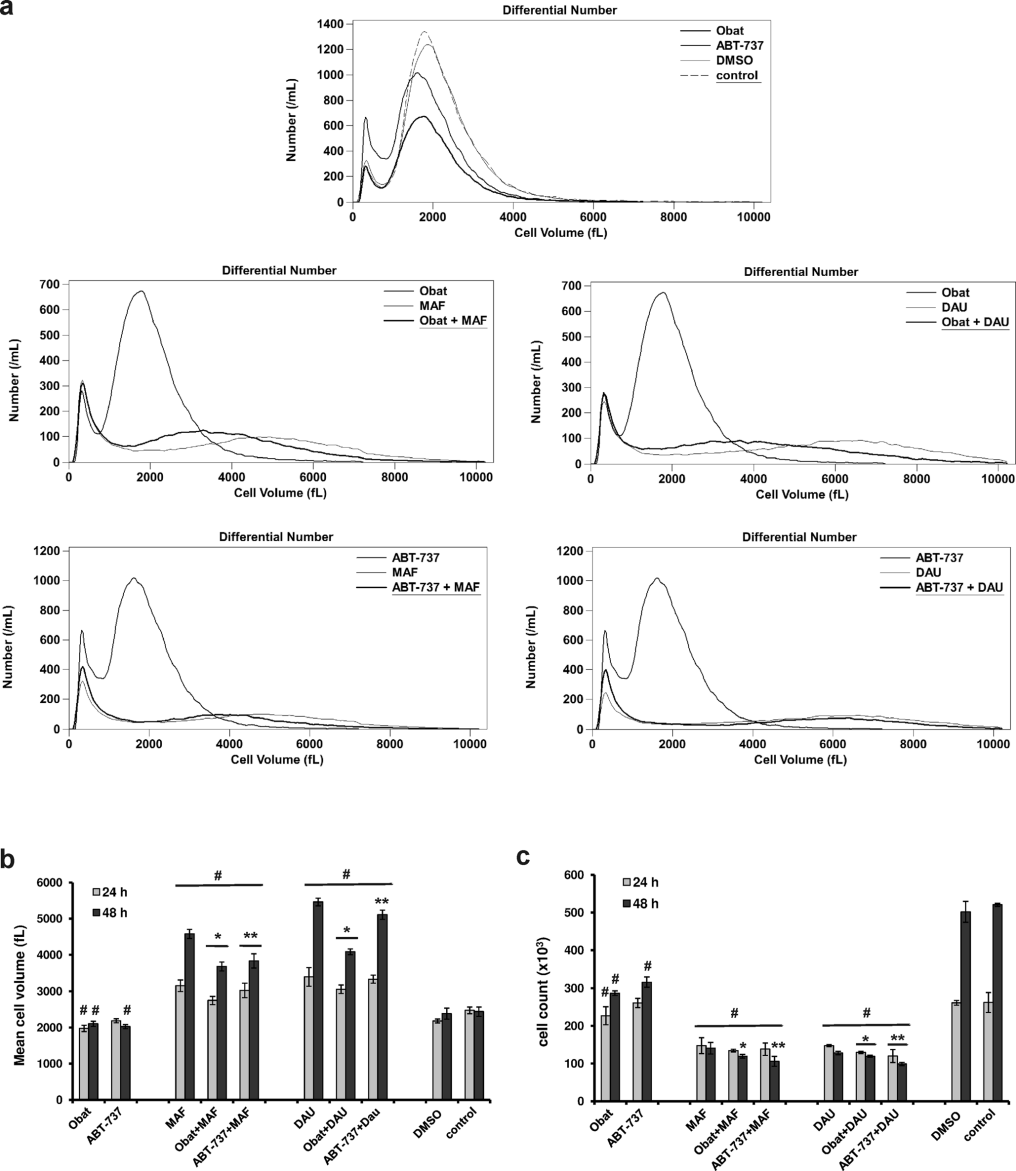
Effects of obatoclax and ABT-737 given alone or in combination with mafosfamide or daunorubicin on the mean volume and count of HL-60 cells, determined using Coulter electrical impedance method. **a** The mean volume distribution curves of HL-60 cells recorded 48 h after cell exposure to the action of Obat (0.5 μM) or ABT-737 (2.5 μM) in combination with MAF (20 μM) or DAU (0.15 μM). The peaks on the left represent cellular debris, presumably apoptotic bodies and necrotic cell fragments, which were excluded from the analysis of the HL-60 cell volume (**b**) and count (**c**). The data are presented as mean ± SD from three independent experiments carried out in duplicate. Values significantly different at *p* < 0.05 according to Duncan’s test: *, ** between a combination group and a single agent-treated group; # compared to control

Cell volume and count are important parameters characterizing cytotoxic activity of anticancer agents. The regulation of cell volume is an essential function that is coupled to a variety of physiological processes including cell proliferation and programmed cell death. It has been shown that functional and morphological alterations occurring in cells following their exposure to cytotoxic agents caused changes in cell volume [17]. As shown in Figure 4, Obat and ABT-737 given alone caused a decrease of the HL-60 leukemia mean cell volume. The observed changes in the leukemia cell volume were found 24 and 48 h after Obat application and at 48 h after the cell exposure to ABT-737. The mean cell volume distinctly increased 24 and 48 h after MAF and DAU application as single agents. The simultaneous exposure of HL-60 cells to Obat and MAF or DAU resulted in a decrease of the mean cell volume compared with the values obtained at the indicated two time intervals after application of mafosfamide and daunorubicin as single agents. The combined treatment of HL-60 cells with ABT-737 and MAF or DAU caused a decrease of the mean cell volume observed at 48 h time point (Fig. 4 b). About 95 % of untreated and DMSO-treated control cells exhibited volumes between 832–3917 fL. MAF and DAU caused a significant increase in cell counts determined at a cell volume range 3917–7346 fL. The lower frequency of HL-60 cells with the volumes ranging from 3917–7346 were observed after the combined action of BH3 mimetic and MAF or DAU as compared with the percentage values found after the single application of MAF and DAU (Table 2).

**Table 2:** Changes in the frequency of HL-60 leukemia cells with the volumes ranging from 3917 to 7346 fL. For each experimental group data were calculated as a percentage of total cell count determined at a cell volume range 832–7346 fL

	HL-60 cells with the volumes >3917 fL (%)									
	Obat	ABT-737	MAF	Obat + MAF	ABT-737 + MAF	DAU	Obat + DAU	ABT-737 + DAU	DMSO	Control
24 h	3.10	4.79	39.44	17.43	28.09	50.56	26.52	48.83	5.09	5.00
48 h	4.37	3.62	64.85	45.21	48.77	72.49	52.80	69.43	6.24	6.65

BH3 mimetic application resulted in a reduction of the HL-60 cell count. The cell count decreased 24 and 48 h after HL-60 cell exposure to the action of obatoclax and 48 h after ABT-737 application. The combined application of Obat or ABT-737 with MAF or DAU affected the leukemia cell count to a higher degree than did each of the tested agents given alone (Fig. 4 c).

### Cell death induction

**Fig. 5:**
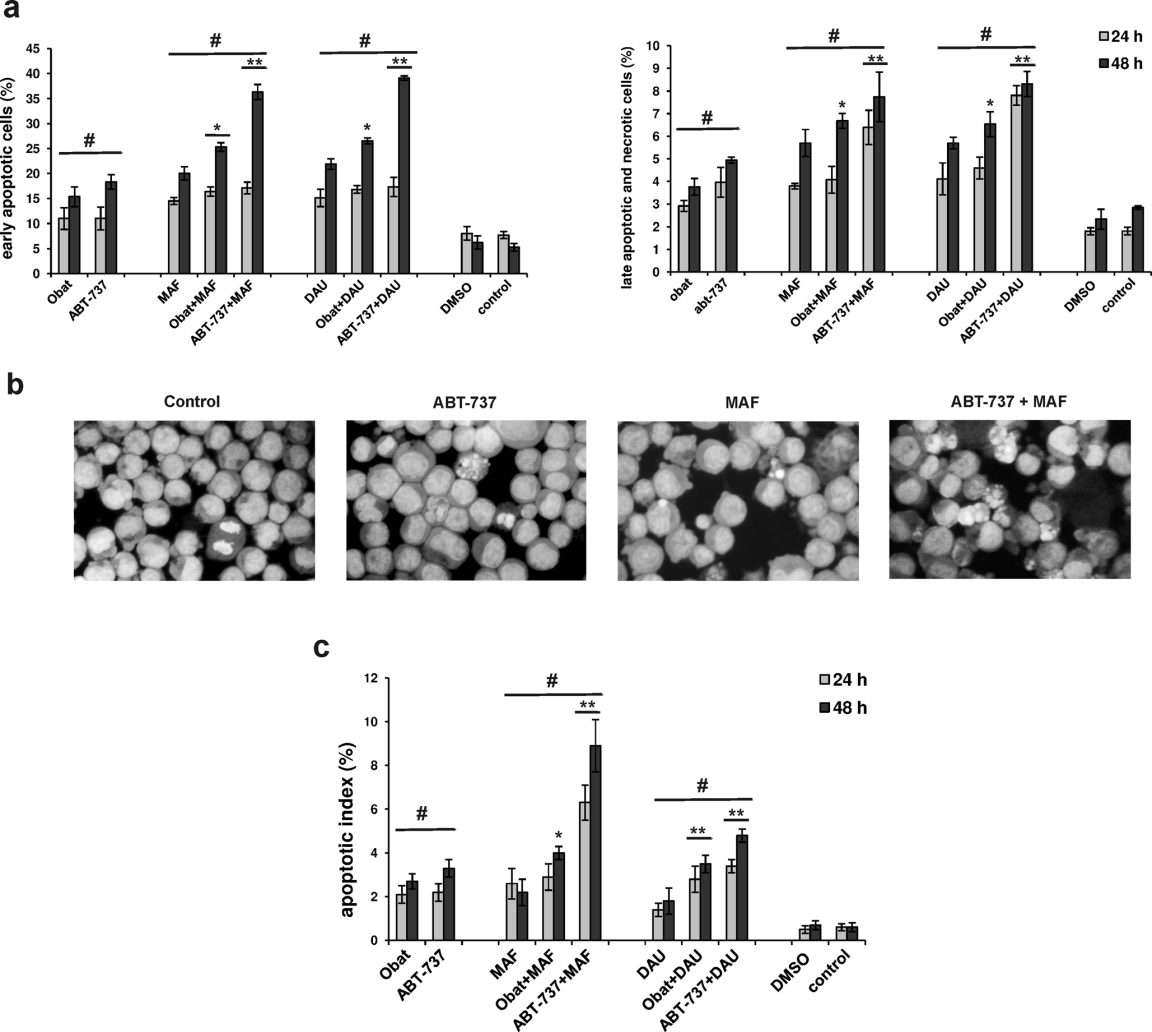
Effects of obatoclax and ABT-737 given alone or in combination with mafosfamide or daunorubicin on apoptosis-induction in HL-60 cells, determined using flow cytometry annexin V-FITC/PI assay and morphological analysis. The leukemia cells were exposed to Obat (0.5 μM) or ABT-737 (2.5 μM) in the absence or presence of MAF (20 μM) or DAU (0.15 μM). **a** The frequency of early apoptotic cells (annexin V-FITC positive/PI negative) and late apoptotic and necrotic cells (annexin V-FITC positive/PI positive). **b** Representative micrographs of HL-60 cells stained with acridine orange, demonstrating cellular shrinkage, chromatin condensation and nuclear fragmentation in apoptotic cells. Original magnification, 400×. **c** Quantitative assessment of apoptotic HL-60 cells carried out under fluorescence microscope. The data are presented as mean ± SD from three independent experiments. Values significantly different at *p* < 0.05 according to Duncan’s test: *, ** between a combination group and a single agent-treated group; # compared to control

The effects of BH3 mimetics used alone or in combination with mafosfamide or daunorubicin on triggering phosphatidylserine externalization, plasma membrane disruption and morphological changes of HL-60 cells, were assessed. Using the flow cytometry fluoresceinated annexin V/propidium iodide assay, the frequency of early apoptotic cells, and the frequency of HL-60 cells undergoing late apoptosis and necrosis, were determined (Fig. 5a). The exposure of HL-60 cells to Obat or ABT-737 given alone significantly triggered apoptosis. The distinctly higher frequency of early apoptotic and frequency of late apoptotic and necrotic cells, were observed after the combined action of BH3 mimetic and MAF or DAU as compared with the percentage values found after the single application of each of the tested agent. The combination of ABT-737 with mafosfamide or daunorubicin was more effective in apoptosis-induction than the combination of obatoclax with these agents.

The microscopic analysis of HL-60 cells stained with acridine orange confirmed that obatoclax and ABT-737 applied alone and with MAF or DAU distinctly increase the frequency of apoptotic HL-60 cells. ABT-737 appeared to trigger apoptosis in HL-60 cells more effectively than did obatoclax, in the case when these agents were applied alone and in combination with MAF or DAU (Fig. 5b, c).

## Discussion

In the present investigations, the antileukemic potential of two BH3 mimetics, obatoclax and ABT-737, given alone and in combination with mafosfamide or daunorubicin, was assessed. Data presented in this study have demonstrated the cytotoxic effects of obatoclax and ABT-737 on HL-60, U-937, ML-1 and MOLT-4 leukemia cells. On the basis of the obtained results using the cell viability assay, it can be stated that the antileukemic activity of ABT-737 was weaker than that of obatoclax. The present study has also shown that obatoclax and ABT-737 can modify the leukemia cell response to the action of MAF and DAU. The combination of BH3 mimetic with anticancer agent caused a synergistic decrease of the leukemia cell viability and significant changes in the cell volume and count. Moreover, the results of flow cytometry and microscopy analysis demonstrated that obatoclax and ABT-737 increased the yield of apoptosis triggered by mafosfamide and daunorubicin in leukemia cells. ABT-737 combined with MAF or DAU induced apoptosis more effectively than obatoclax did in the same combination regimen. To our knowledge, the findings of the present study are the first data which compare the antileukemic potential of obatoclax and ABT-737 used as a single agent and in combination with such anticancer agents as MAF and DAU.

Differences between the cytotoxic activities of obatoclax and ABT-737 found in the present study may depend on the expression profile of anti-apoptotic Bcl-2 proteins in leukemia cell lines. The literature data clearly indicate the affinity of obatoclax and ABT-737 to Bcl-2 subfamily members. Obatoclax has been shown to uniformly inhibit all of the anti-apoptotic Bcl-2 family proteins, Bcl-2, Bcl-xL, Mcl-1, Bcl-w, and A1 [18, 19]. ABT-737 acts selectively for Bcl-2, Bcl-xL, and Bcl-w but not for Mcl-1. Studies demonstrated that sensitivity to ABT-737 is decreased in cells expressing elevated level of Mcl-1 [20, 21]. In the present study, ML-1 and U937 cells were found to be more resistant to ABT-737 than HL-60 and MOLT-4 cells. ML-1 and U937 cells express higher levels of Mcl-1 protein whereas Bcl-2 is overexpressed in HL-60 cells [22–26]. MOLT-4 cells were found to have a high level of Bcl-xL [26, 27]. It is also possible that obatoclax and ABT-737 – induced cytotoxicity may depend not only on expression of anti-apoptotic proteins of Bcl-2 family but also on pro-apoptotic protein expression [28] and/or p53 status [29].

Differences in the antileukemic activities of obatoclax and ABT-737 can be also related to their mechanisms of action, which are still incompletely explained. Recent studies have shown that the cytotoxic effects of obatoclax and ABT-737 on leukemia cells result from apoptosis-induction [19, 20, 28, 30]. In obatoclax-treated cells, the liberation of Bak from Mcl-1, dissociation of Bim from Bcl-2 and Mcl-1, and the formation of a complex of Bak with Bax, were found [19]. Obatoclax also acts as a direct activating stimulus for Bax activation [31]. Mechanistic studies revealed that ABT-737 is similar to the BH3 domain of Bad. By itself ABT-737 does not bind to Bax, but disrupts the complex of Bax and Bcl-2 and triggers conformational alteration of Bax [30]. ABT-737 also displaces BH3-only proteins such as Bim from the BH3-binding pocket of Bcl-2, allowing Bim to activate Bax and induce mitochondrial membrane permeabilization [32]. In the study of Vogler et al. [28], it was shown that between six putative Bcl-2 inhibitors, including obatoclax, ABT-737 and gossypol, only ABT-737 was unable to kill Bax/Bak double knockout cells. It was found that apoptosis induced by obatoclax was diminished, but not abolished, in the absence of Bak/Bax, suggesting that additional target(s) other than Bcl-2 contribute to the activation of the mitochondrial pathway by this agent. Some recent reports have demonstrated that obatoclax caused the cell cycle arrest [19, 33], necroptosis [34–36] and death-promoting autophagy [37] in leukemia cells. The activation of alternative cell death pathways by obatoclax is a possible explanation for its stronger cytotoxic effects on the leukemia cell viability than that observed for ABT-737.

Recent in vitro and in vivo studies on solid tumors and hematological malignancies have shown that obatoclax and ABT-737 significantly potentiate the anticancer efficacy of established and novel chemotherapeutic drugs [27, 30, 38–41]. The results obtained in the present investigation strengthened the preclinical evidence of significant antileukemic activity of obatoclax and ABT-737 in combinations with novel anticancer agents and chemotherapeutic drugs. In the present study, the leukemia cells were exposed to the action of BH3 mimetics in combination with mafosfamide and daunorubicin. It was shown that MAF and DAU induced apoptosis in different types of cancer cells [42–45]. The mechanisms of mafosfamide- and daunorubicin- induced apoptosis have not been fully characterized. The anticancer effects of MAF and DAU are generally considered to originate from damage to DNA [42, 46]. DNA replication blockage followed by p53 activation appeared to trigger apoptosis following mafosfamide treatment in lymphoblastoid cells. A less efficient p53 independent pathway resulting in Bcl-2 decline can also be activated in response to mafosfamide [43]. Daunorubicin functions by inhibiting topoisomerase II, but also have the potential to form lethal DNA adducts in cancer cells leading to apoptosis [42]. It has been shown that another anthracycline, doxorubicin, increased the amount of Mcl-1/Noxa complexes in U937 cells. The association between Mcl-1 and Noxa favors Mcl-1 degradation, reducing its stability through a conformational change [47]. Previous studies from our laboratory have shown that mafosfamide and daunorubicin may trigger apoptosis through mitochondrial pathway [44, 45].

The synergistic interactions between BH3 mimetics and anticancer agents observed in the present study may be explained by the distinct but complementary mechanisms of activation of the mitochondrial pathway of apoptosis. The combination of BH3-mimetics with DNA-damaging agents may achieve greater inhibition of Bcl-2 proteins than the application of either agent alone. In the study of Mason et al. [48] the synergy between ABT-737 and low-dose cyclophosphamide in mice transplanted with myc/bcl-2 lymphomas was demonstrated. Cyclophosphamide caused activation of p53 followed by neutralization of Mcl-1 and enhanced apoptosis induced by ABT-737. The ability of ABT-737 to increase the antileukemic activity of daunorubicin has been shown previously in acute lymphoblastic leukemia cells with MLL rearrangement [49]. Recently, Ugarenko et al. [22] have demonstrated that ABT-737 enhances the antileukemic activity of doxorubicin-DNA adducts by the Bcl-2 inhibition. The results obtained in the present investigation are in agreement with previous studies showing that ABT-737 acts synergistically with other anticancer agents to induce apoptosis in cancer cells. It was found that ABT-737 induced apoptosis more effectively than did obatoclax when these BH3 mimetics were combined with cytotoxic agents. It is interesting to note that obatoclax exhibited antileukemic activity in lower concentration than did ABT-737, as shown by the IC_50_ values, and synergized with anticancer agents to reduce the leukemia cell viability. It can be assumed that not only apoptosis-induction but also other processes occurring in the leukemia cells may be involved in the synergistic decrease of their viability observed after the combined application of obatoclax and DNA-damaging agents. In previous studies, it has been shown that antileukemic activity of mafosfamide and daunorubicin could be manifested by cell cycle disruption, cell proliferation inhibition, mitotic catastrophe, and necrosis induction [15, 45, 50, 51]. In the present study, the increase of cell volume was observed in HL-60 cell exposed to MAF and DAU and the decrease of mean cell volume was found after BH3 mimetic application. It is assumed that an increase of the mean cell volume may indicate that the cells undergo mitotic catastrophe or programmed necrosis, and a decrease of the mean cell volume can be the result of apoptotic processes occurring in the cell population [17, 52]. Further research should be conducted to clarify precisely the reason of volume changes in leukemia cells exposed to the obatoclax and ABT-737 alone and in combination with DNA-damaging agents.

In conclusion, both BH3 mimetics obatoclax and ABT-737, have high antileukemic activity and significantly potentiate the efficacy of the oxazaphosphorine and anthracycline agents. The obtained results have clearly shown that obatoclax and ABT-737 are promising agents for the treatment of leukemia. Nevertheless, the differences in antileukemic potential of obatoclax and ABT-737 applied alone and in combination with DNA-damaging agents should be further explored.
